# Supplementary material for: Isotope engineering achieved by local coordination design in Ti-Pd co-doped ZrCo-based alloys
Source: Nat Commun. 2024 Apr 3;15:2883. doi: 10.1038/s41467-024-47250-3 (PMC10991433; doi:10.1038/s41467-024-47250-3)
Supplement: Supplementary file 1 — Supplementary Information [file 41467_2024_47250_MOESM1_ESM.pdf]

Supplementary Information

Isotope engineering achieved by local coordination design in Ti-Pd co-doped ZrCo-based alloys

Jiacheng Qi,[1,†] Xu Huang,[2,†] Xuezhang Xiao,[1,3,*] Xinyi Zhang,[1] Panpan Zhou,[1] Shuoqing Zhang,[1] Ruhong Li,[4] Huaqin Kou,[2,*] Fei Jiang,[2] Yong Yao,[2] Jiangfeng Song,[2] Xingwen Feng,[2] Yan Shi,[2] Wenhua Luo,[2] and Lixin Chen[1,*]

[1]State Key Laboratory of Silicon and Advanced Semiconductor Materials, School of Materials Science and Engineering, Zhejiang University, Hangzhou 310058, Zhejiang, China. E-mail: xzxiao@zju.edu.cn; lxchen@zju.edu.cn

[2]Institute of Materials, China Academy of Engineering Physics, Mianyang 621907, Sichuan, China. E-mail: kouhuaqin@caep.cn

[3]Key Laboratory of Hydrogen Storage and Transportation Technology of Zhejiang Province, Hangzhou 310027, Zhejiang, China.

[4]ZJU-Hangzhou Global Scientific and Technological Innovation Center, Zhejiang University, Hangzhou 311215, China.

[†]These authors contributed equally to this work.

Inventory of Supporting Information

Supplementary Table 1 List of abbreviations and their corresponding detailed description.

Supplementary Table 2 Relative energy difference for ZrCo alloy during protium/deuterium adsorption, dissociation and diffusion processes.

Supplementary Table 3 Screening results for $T_{1\ \mathrm{bar}}$ and T_{cr} of $Zr_{0.75}A_{0.25}Co$ (A=Sc, Ti, V, Y, Zr, Nb, Hf) with Zr site doping and $ZrCo_{0.75}B_{0.25}$ (B=Cr, Mn, Fe, Co, Ni, Cu, Zn, Mo, Ru, Rh, Pd, Ag, Cd) with Co site doping.

Supplementary Table 4 Cumulative hydrogen concentration of release gas at certain temperatures during the whole desorption process.

Supplementary Table 5 Thermodynamic parameters for protium/deuterium absorption processes of $Zr_{0.8}Ti_{0.2}Co_{0.8}Pd_{0.2}$ alloy.

Supplementary Table 6 Thermodynamic parameters for protium/deuterium absorption and desorption processes of $ZrCo_{0.8}Pd_{0.2}$ alloy.

Supplementary Fig. 1 Summary of thermodynamic parameters of ZrCo-based alloys. Solid and open legends represent hydrogen isotope absorption and desorption processes, respectively. Rectangle and circle legends mean protium and deuterium, respectively.

Supplementary Fig. 2 XRD patterns of ZrCo alloy at different temperatures during TPD.

Supplementary Fig. 3 Kissinger fitting curves of ZrCo hydride and deuteride. Rectangle and circle legends mean hydride and deuteride, respectively.

Supplementary Fig. 4 PCI curves of ZrCo alloy. Solid and open legends represent deuterium absorption and desorption processes, respectively.

Supplementary Fig. 5 PCI curves of $Zr_{0.8}Ti_{0.2}Co$ alloy. Solid and open legends represent deuterium absorption and desorption processes, respectively.

Supplementary Fig. 6 XRD patterns of $Zr_{0.8}Ti_{0.2}Co$ alloy at different hydrogen pressures during absorption and desorption processes.

Supplementary Fig. 7 DSC curves of $Zr_{0.8}Ti_{0.2}Co$ hydride and deuteride.

Supplementary Fig. 8 XRD patterns of $Zr_{0.8}Ti_{0.2}Co$ alloy at different temperatures during thermal desorption.

Supplementary Fig. 9 XRD patterns of $Zr_{0.8}Ti_{0.2}Co_{0.8}Pd_{0.2}$ hydride and deuteride.

Supplementary Fig. 10 Kissinger fitting curves of $Zr_{0.8}Ti_{0.2}Co_{0.8}Pd_{0.2}$ hydride and deuteride. Rectangle and circle legends mean hydride and deuteride, respectively.

Supplementary Fig. 11 TPD profiles of ZrCo, $Zr_{0.8}Ti_{0.2}Co$ and $Zr_{0.8}Ti_{0.2}Co_{0.8}Pd_{0.2}$ hydrides with the heating rate of 5 °C/min.

Supplementary Fig. 12 TDS curves of ZrCo sample saturated in the mixed gas with H_2/D_2=1/1.

Supplementary Fig. 13 TDS curves of $Zr_{0.8}Ti_{0.2}Co$ sample saturated in the mixed gas with H_2/D_2=1/1.

Supplementary Fig. 14 PCI curves of $Zr_{0.8}Ti_{0.2}Co_{0.8}Pd_{0.2}$ alloy for protium absorption.

Supplementary Fig. 15 PCI curves of $Zr_{0.8}Ti_{0.2}Co_{0.8}Pd_{0.2}$ alloy for deuterium absorption.

Supplementary Fig. 16 PCI curves of $Zr_{0.8}Ti_{0.2}Co_{0.8}Pd_{0.2}$ alloy for protium desorption.

Supplementary Fig. 17 PCI curves of $Zr_{0.8}Ti_{0.2}Co_{0.8}Pd_{0.2}$ alloy for deuterium desorption.

Supplementary Fig. 18 XRD pattern and Rietveld refinement result of $ZrCo_{0.8}Pd_{0.2}$ alloy.

Supplementary Fig. 19 Characterization of $ZrCo_{0.8}Pd_{0.2}$ alloy. a SEM image, **b** TEM and corresponding HRTEM images, **c** STEM-HAADF image with EDS, **d** Quantitative content of alloy elements of $ZrCo_{0.8}Pd_{0.2}$ alloy.

Supplementary Fig. 20 PCI curves of $ZrCo_{0.8}Pd_{0.2}$ alloy for protium absorption.

Supplementary Fig. 21 PCI curves of $ZrCo_{0.8}Pd_{0.2}$ alloy for deuterium absorption.

Supplementary Fig. 22 PCI curves of $ZrCo_{0.8}Pd_{0.2}$ alloy for protium desorption.

Supplementary Fig. 23 PCI curves of $ZrCo_{0.8}Pd_{0.2}$ alloy for deuterium desorption.

Supplementary Fig. 24 Cycling process parameters. Cyclic system pressures **a** and corresponding temperatures **b** for absorption and desorption processes.

Supplementary Fig. 25 Cycling performance and characterization of ZrCo, $Zr_{0.8}Ti_{0.2}Co$ and $Zr_{0.8}Ti_{0.2}Co_{0.8}Pd_{0.2}$ alloys. Cyclic deuterium absorption **a** and desorption **b** capacities, and corresponding XRD patterns of deuterides **c** and alloys **d** at 50th cycle.

Supplementary Note 1 The expression of separation factor

Separation factor can be calculated by the ratio of square root of plateau pressure for single-isotope isotherms.

$$\alpha = \sqrt{\frac{P_{Q_l}}{P_{Q_h}}} \quad (1)$$

where P_{Q_l} and P_{Q_h} represent the plateau pressure of isotherms for single light and heavy isotopes, respectively.

$$\ln P_{eq} = -\frac{\Delta H}{RT} + \frac{\Delta S}{R} \quad (2)$$

where P_{eq} means the plateau pressure, ΔH and ΔS represent the enthalpy and entropy change of the reaction ($\frac{2}{x}MQ_x \leftrightarrow \frac{2}{x}M + Q_2$), R is the gas constant (8.314 $J \cdot mol^{-1} \cdot K^{-1}$) and T represents temperature. Combining the Eq. (1) and (2), the separation factor can be expressed by

$$\alpha = e^{-\frac{1}{2RT}\left(\Delta H_{Q_l} - \Delta H_{Q_h}\right) + \frac{1}{2R}\left(\Delta S_{Q_l} - \Delta S_{Q_h}\right)} \quad (3)$$

Supplementary Note 2 The expression of harmonic oscillator model

The temperature-dependent partition function of gaseous molecules can be expressed as

$$Z_{\text{gas}} = \exp\left[\sum_{n=0}^{4} a_n \left(\frac{300}{T}\right)^n\right] \tag{4}$$

where a_0, a_1, a_2, a_3 and a_4 are 0.89156, 1.36456, -0.00075, -0.00625 and 0.00638, respectively. Based on the harmonic oscillator model (Eq. (5) and (6)), partition function for dissolved hydrogen isotopes depends on the local vibration mode dictated by temperature and interstitial environment.

$$Z_{\text{solid}} = \left\{\frac{\sinh(u_{\text{A}}/2)}{\sinh\left[u_{\text{A}}/2(m_{\text{B}}/m_{\text{A}})^{1/2}\right]}\right\}^3 \tag{5}$$

$$u = \frac{\hbar\omega}{k_{\text{B}}T} \tag{6}$$

where u_{A} means reduced temperature of light hydrogen isotope, m_{A} and m_{B} are the mass of light and heavy isotopes, respectively, k_{B} represents Boltzmann constant, $\hbar$ represents reduced Planck constant, ω is local vibration frequency and T is temperature.

Supplementary Tables

Supplementary Table 1 List of abbreviations and their corresponding detailed description.

Abbreviation	Detailed description
IFC	Inner fuel cycle
TEP	Tokamak exhaust processing system
ISS	Isotope separation system
SDS	Storage and delivery system
PCI	Pressure-composition isotherm
HMIE	Homomolecular isotope exchange reaction
ZPE	Zero-point energy
HID	Hydrogen-induced disproportionation reaction
DU	Depleted uranium
H	Protium
D	Deuterium
T	Tritium
Q	Hydrogen isotope
DSC	Differential scanning calorimetry
TPD	Temperature programmed desorption
TDS	Thermal desorption spectroscopy
α	Separation factor
Q_l	Atomic fraction of the light isotope in a certain phase
Q_h	Atomic fraction of the heavy isotope in a certain phase
T_{cr}	Critical temperature for zero isotope effect
$T_{1\ bar}$	Operating temperature for atmospheric pressure delivery
T_{gap}	Mismatch between T_{cr} and $T_{1\ bar}$
E_a	Apparent activation energy for the desorption process
T_p	Peak temperature of DSC curve
β	Heating rate
A	Pre-exponential factor
P_{eq}	Equilibrium plateau pressure
ΔH	Enthalpy change
ΔS	Entropy change
Z	Partition function for hydrogen isotopes in a certain phase

ω_H	Vibration frequency of interstitial hydrogen
$\hbar$	Reduced Planck constant

Supplementary Table 2 Relative energy difference for ZrCo alloy during protium/deuterium adsorption, dissociation and diffusion processes.

Reaction coordinate	Relative energy (eV)	
	Q=H	Q=D
Q_2	0.00	0.00
Q_2 (Zr-T site)	-0.19	-0.22
TS1	0.17	0.19
Q (Zr-B site)	-0.87	-0.88
Q (ZrZrCo-H site)	-1.53	-1.55
TS2	-0.77	-0.79
Q (subsurface)	-0.92	-0.94
TS3	-0.35	-0.38
Q (bulk)	-1.11	-1.13

Supplementary Table 3 Screening results for $T_{1\ bar}$ and T_{cr} of $Zr_{0.75}A_{0.25}Co$ (A=Sc, Ti, V, Y, Zr, Nb, Hf) with Zr site doping and $ZrCo_{0.75}B_{0.25}$ (B=Cr, Mn, Fe, Co, Ni, Cu, Zn, Mo, Ru, Rh, Pd, Ag, Cd) with Co site doping.

Alloy Composition	Average $T_{1\ bar}$ (°C)	Average T_{cr} (°C)	T_{gap} (°C)
ZrCo	420.74	159.97	260.77
$Zr_{0.75}Sc_{0.25}Co$	430.73	255.70	175.03
$Zr_{0.75}Ti_{0.25}Co$	354.58	209.79	144.79
$Zr_{0.75}V_{0.25}Co$	299.35	82.11	217.24
$Zr_{0.75}Y_{0.25}Co$	484.02	149.73	334.29
$Zr_{0.75}Nb_{0.25}Co$	363.59	72.01	291.58
$Zr_{0.75}Hf_{0.25}Co$	398.96	154.27	244.69
$ZrCo_{0.75}Cr_{0.25}$	417.57	175.69	241.88
$ZrCo_{0.75}Mn_{0.25}$	415.67	238.76	176.91
$ZrCo_{0.75}Fe_{0.25}$	430.40	224.11	206.29
$ZrCo_{0.75}Ni_{0.25}$	451.09	82.20	368.89
$ZrCo_{0.75}Cu_{0.25}$	439.79	91.30	348.49
$ZrCo_{0.75}Zn_{0.25}$	394.50	65.60	328.9
$ZrCo_{0.75}Mo_{0.25}$	329.04	97.59	231.45
$ZrCo_{0.75}Ru_{0.25}$	340.89	139.74	201.15
$ZrCo_{0.75}Rh_{0.25}$	347.31	114.04	233.27
$ZrCo_{0.75}Pd_{0.25}$	377.06	213.27	163.79
$ZrCo_{0.75}Ag_{0.25}$	379.59	172.99	206.6
$ZrCo_{0.75}Cd_{0.25}$	344.53	126.93	217.6

Supplementary Table 4 Cumulative hydrogen concentration of release gas at certain temperatures during the whole desorption process.

Temperature (°C)	Hydrogen concentration (%)		
	ZrCo	$Zr_{0.8}Ti_{0.2}Co$	$Zr_{0.8}Ti_{0.2}Co_{0.8}Pd_{0.2}$
150	54.44	53.55	53.10
200	53.78	53.00	52.28
250	53.34	52.27	50.21
300	51.92	51.51	49.26
350	50.02	49.85	49.24
400	49.93	49.52	49.40
450	49.98	49.67	49.60
500	50.05	49.82	49.60
550	50.07	49.85	49.62
600	50.05	50.02	49.82
650	50.04	50.01	50.02

Supplementary Table 5 Thermodynamic parameters for protium/deuterium absorption processes of $Zr_{0.8}Ti_{0.2}Co_{0.8}Pd_{0.2}$ alloy.

Parameters	Absorption			
	Low plateau		High plateau	
	Q=H	Q=D	Q=H	Q=D
ΔH (kJ·mol^{-1} Q_2)	-66.87	-67.44	-47.00	-54.90
ΔS (J·K^{-1}·mol^{-1} Q_2)	-115.90	-118.97	-105.91	-117.72

Supplementary Table 6 Thermodynamic parameters for protium/deuterium absorption and desorption processes of $ZrCo_{0.8}Pd_{0.2}$ alloy.

Parameters	Absorption		Desorption	
	Q=H	Q=D	Q=H	Q=D
ΔH (kJ·mol^{-1} Q_2)	-84.14	-88.74	85.72	89.25
ΔS (J·K^{-1}·mol^{-1} Q_2)	-133.83	-143.77	129.15	136.72
$T_{1\ bar}$ (° C)	/	/	390.72	379.79
T_{cr} (° C)	189.78		193.31	
T_{gap} (° C)	/		251.62	

Supplementary Figures

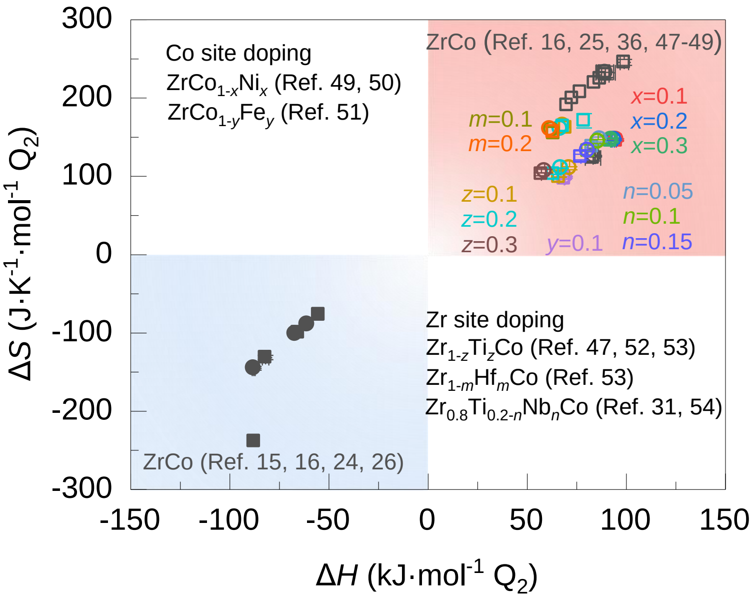

Supplementary Fig. 1 Summary of thermodynamic parameters of ZrCo-based alloys. Solid and open legends represent hydrogen isotope absorption and desorption processes, respectively. Rectangle and circle legends mean protium and deuterium, respectively.

Supplementary Fig. 1 displays the thermodynamic parameters of ZrCo-based alloys previously reported for hydrogen isotope absorption and desorption processes. The inconsistency of thermodynamic parameters hinders one to obtain the accurate thermodynamic isotope effect of ZrCo-based alloys.

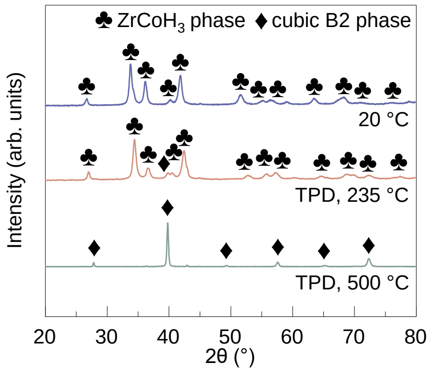

Supplementary Fig. 2 XRD patterns of ZrCo alloy at different temperatures during TPD.

Supplementary Fig. 2 displays the XRD patterns of ZrCo alloy at different temperatures during TPD. The phase component maintains the orthorhombic $ZrCoH_3$ phase and transforms into the cubic B2 phase when temperature reaches 235 °C and 500 °C, which validates the desorption pathway.

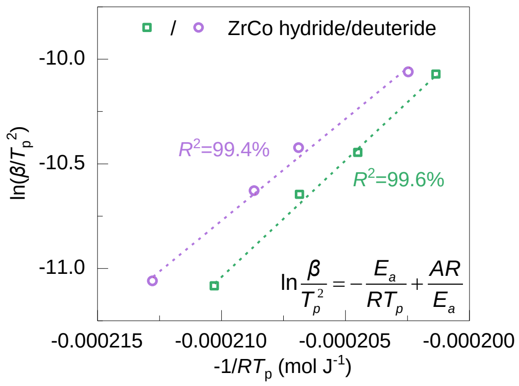

Supplementary Fig. 3 Kissinger fitting curves of ZrCo hydride and deuteride. Rectangle and circle legends mean hydride and deuteride, respectively.

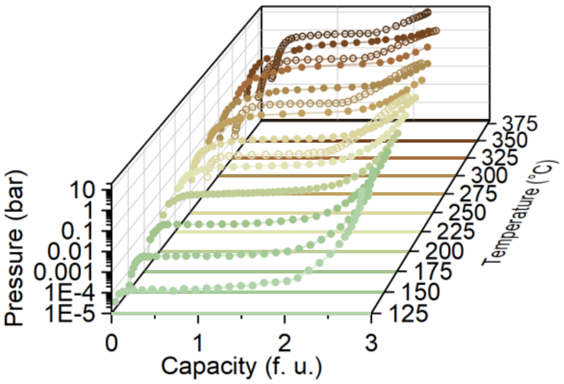

Supplementary Fig. 4 PCI curves of ZrCo alloy. Solid and open legends represent deuterium absorption and desorption processes, respectively.

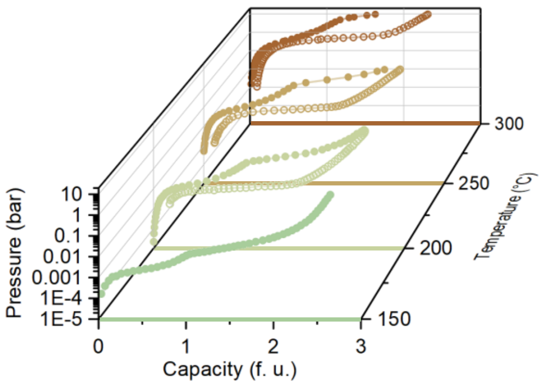

Supplementary Fig. 5 PCI curves of $Zr_{0.8}Ti_{0.2}Co$ alloy. Solid and open legends represent deuterium absorption and desorption processes, respectively.

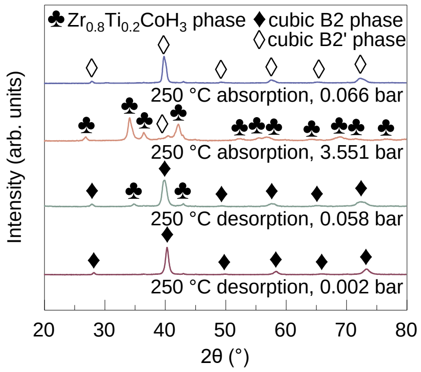

Supplementary Fig. 6 XRD patterns of $Zr_{0.8}Ti_{0.2}Co$ alloy at different hydrogen pressures during absorption and desorption processes.

Supplementary Fig. 6 illustrates the XRD patterns of $Zr_{0.8}Ti_{0.2}Co$ alloy at different hydrogen pressures during absorption and desorption processes. The cubic B2′ phase forms followed by the orthorhombic $Zr_{0.8}Ti_{0.2}CoH_3$ phase during absorption and the orthorhombic $Zr_{0.8}Ti_{0.2}CoH_3$ phase transforms into the cubic B2 phase by one step during desorption, which verifies the absorption-desorption asymmetry.

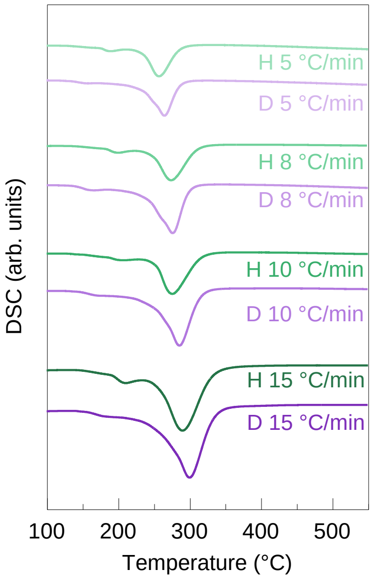

Supplementary Fig. 7 DSC curves of $Zr_{0.8}Ti_{0.2}Co$ hydride and deuteride.

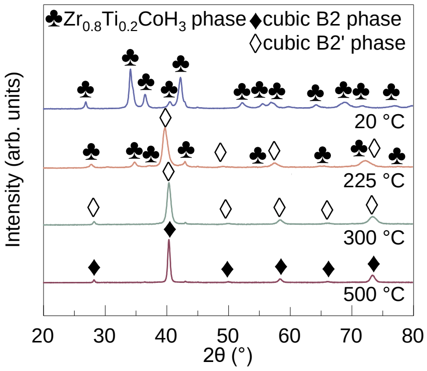

Supplementary Fig. 8 XRD patterns of $Zr_{0.8}Ti_{0.2}Co$ alloy at different temperatures during thermal desorption.

According to the XRD patterns of $Zr_{0.8}Ti_{0.2}Co$ alloy at different temperatures during thermal desorption, the desorption process can be determined to be from the orthorhombic $Zr_{0.8}Ti_{0.2}CoH_3$ phase to the cubic B2′ phase and the cubic B2 phase.

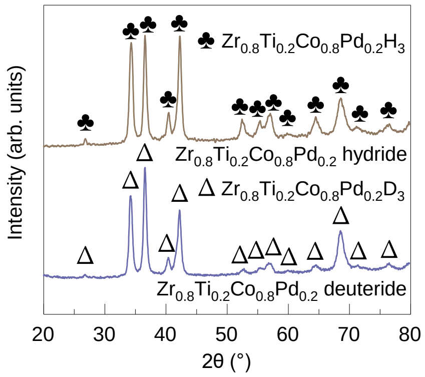

Supplementary Fig. 9 XRD patterns of $Zr_{0.8}Ti_{0.2}Co_{0.8}Pd_{0.2}$ hydride and deuteride.

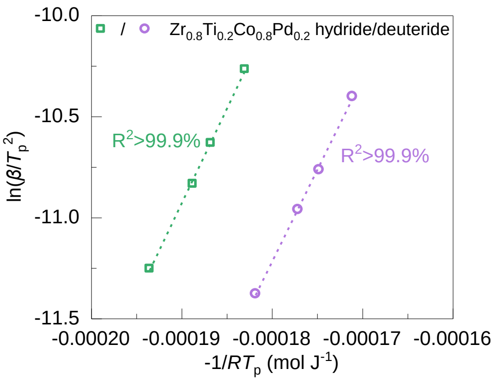

Supplementary Fig. 10 Kissinger fitting curves of $Zr_{0.8}Ti_{0.2}Co_{0.8}Pd_{0.2}$ hydride and deuteride. Rectangle and circle legends mean hydride and deuteride, respectively.

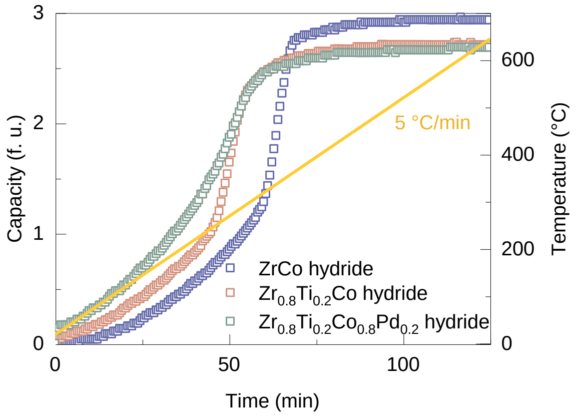

Supplementary Fig. 11 TPD profiles of ZrCo, $Zr_{0.8}Ti_{0.2}Co$ and $Zr_{0.8}Ti_{0.2}Co_{0.8}Pd_{0.2}$ hydrides with the heating rate of 5 °C/min.

Supplementary Fig. 11 illustrates the increasingly enhanced desorption kinetic performance of ZrCo, $Zr_{0.8}Ti_{0.2}Co$ and $Zr_{0.8}Ti_{0.2}Co_{0.8}Pd_{0.2}$ hydrides.

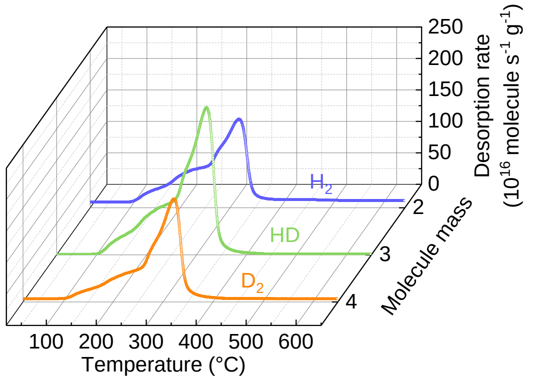

Supplementary Fig. 12 TDS curves of ZrCo sample saturated in the mixed gas with H_2/D_2=1/1.

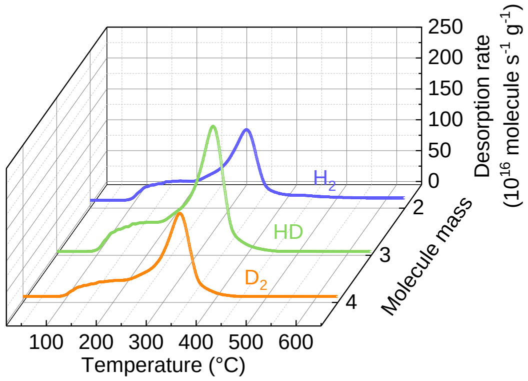

Supplementary Fig. 13 TDS curves of $Zr_{0.8}Ti_{0.2}Co$ sample saturated in the mixed gas with $H_2/D_2=1/1$.

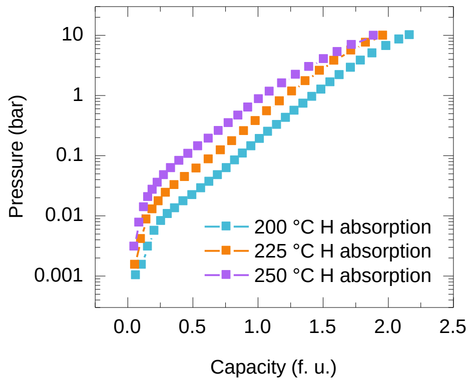

Supplementary Fig. 14 PCI curves of $Zr_{0.8}Ti_{0.2}Co_{0.8}Pd_{0.2}$ alloy for protium absorption.

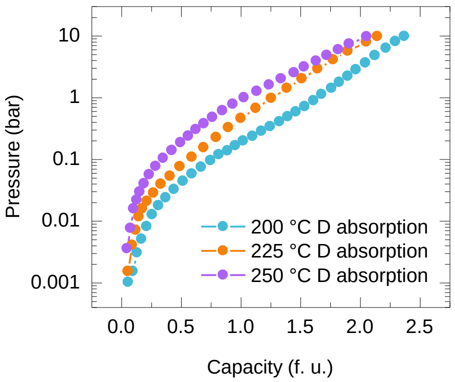

Supplementary Fig. 15 PCI curves of $Zr_{0.8}Ti_{0.2}Co_{0.8}Pd_{0.2}$ alloy for deuterium absorption.

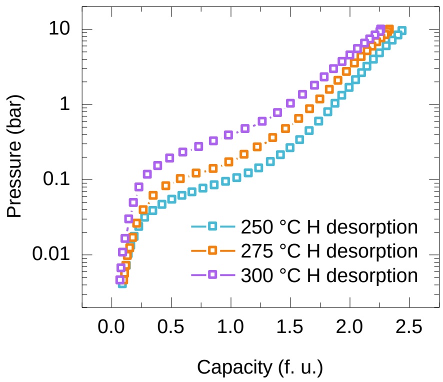

Supplementary Fig. 16 PCI curves of $Zr_{0.8}Ti_{0.2}Co_{0.8}Pd_{0.2}$ alloy for protium desorption.

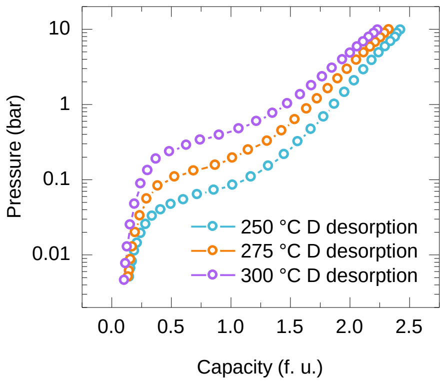

Supplementary Fig. 17 PCI curves of $Zr_{0.8}Ti_{0.2}Co_{0.8}Pd_{0.2}$ alloy for deuterium desorption.

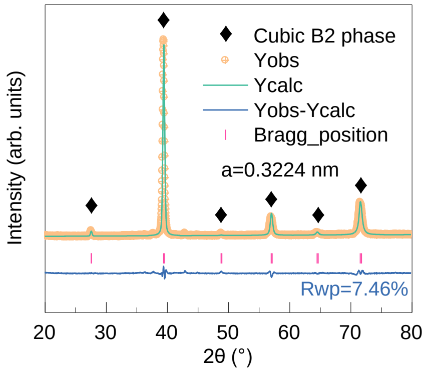

Supplementary Fig. 18 XRD pattern and Rietveld refinement result of $ZrCo_{0.8}Pd_{0.2}$ alloy.

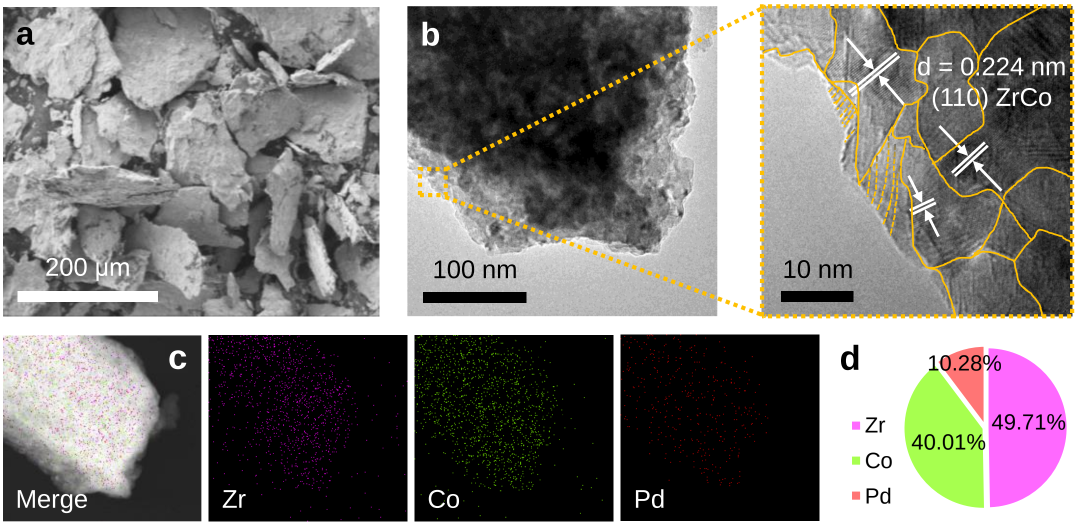

Supplementary Fig. 19 Characterization of $ZrCo_{0.8}Pd_{0.2}$ alloy. **a** SEM image, **b** TEM and corresponding HRTEM images, **c** STEM-HAADF image with EDS, **d** Quantitative content of alloy elements of $ZrCo_{0.8}Pd_{0.2}$ alloy.

Supplementary Fig. 19a and b illustrate the layered microstructure at the micro/nanometer scales and preferred orientation of close-packed crystal plane (110) of $ZrCo_{0.8}Pd_{0.2}$ alloy. Supplementary Fig. 19c and d demonstrate the chemical homogeneity over interlayer and bulk regions and chemical composition of $ZrCo_{0.8}Pd_{0.2}$ alloy.

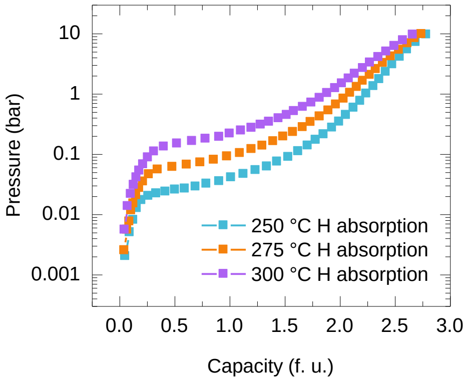

Supplementary Fig. 20 PCI curves of $ZrCo_{0.8}Pd_{0.2}$ alloy for protium absorption.

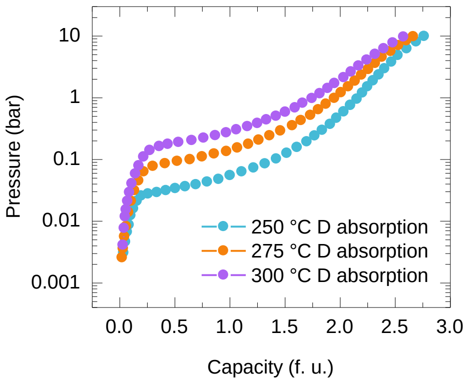

Supplementary Fig. 21 PCI curves of $ZrCo_{0.8}Pd_{0.2}$ alloy for deuterium absorption.

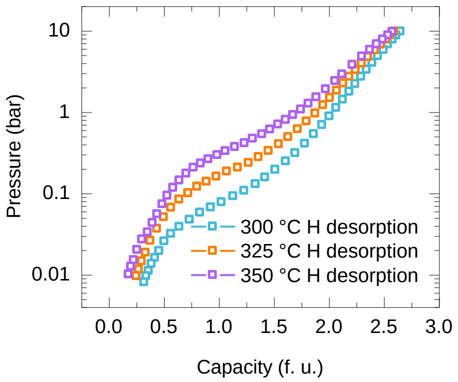

Supplementary Fig. 22 PCI curves of $ZrCo_{0.8}Pd_{0.2}$ alloy for protium desorption.

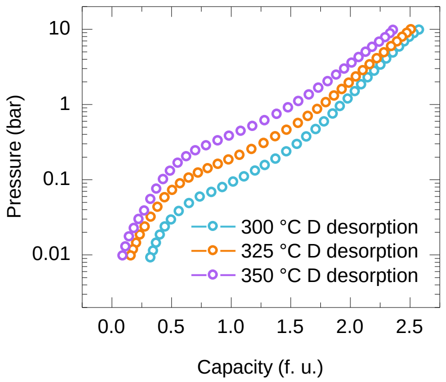

Supplementary Fig. 23 PCI curves of $ZrCo_{0.8}Pd_{0.2}$ alloy for deuterium desorption.

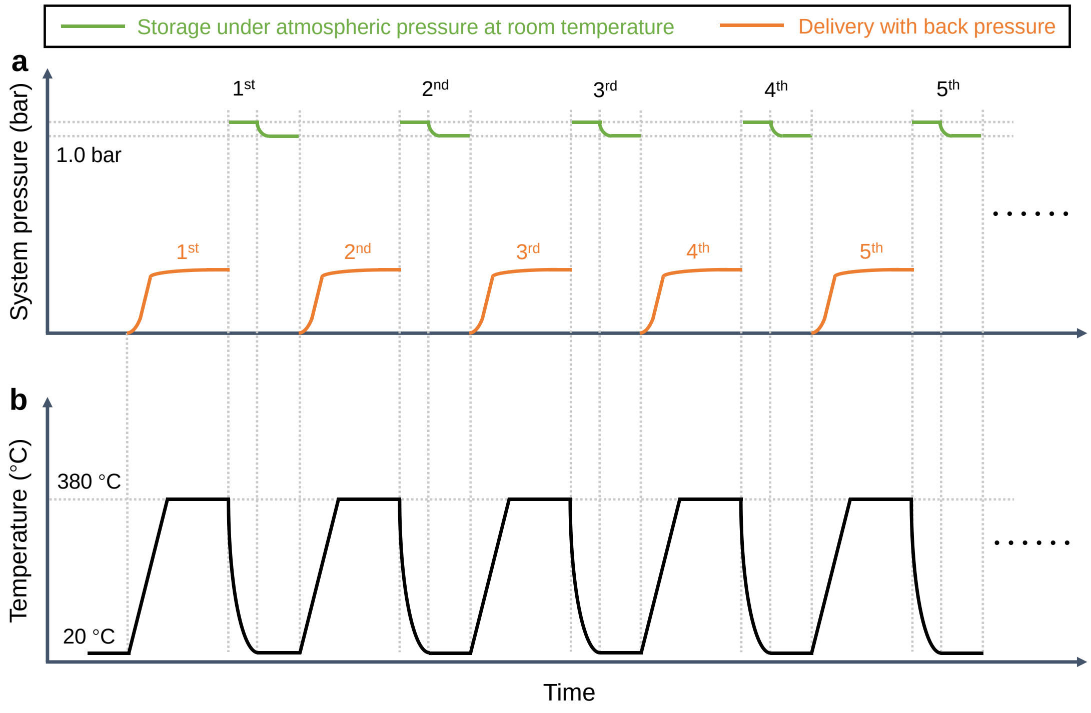

Supplementary Fig. 24 Cycling process parameters. Cyclic system pressures **a** and corresponding temperatures **b** for absorption and desorption processes.

Cycling tests consist of deuterium absorption under atmospheric pressure at room temperature and temperature programmed desorption with deuterium from room temperature to 380 °C.

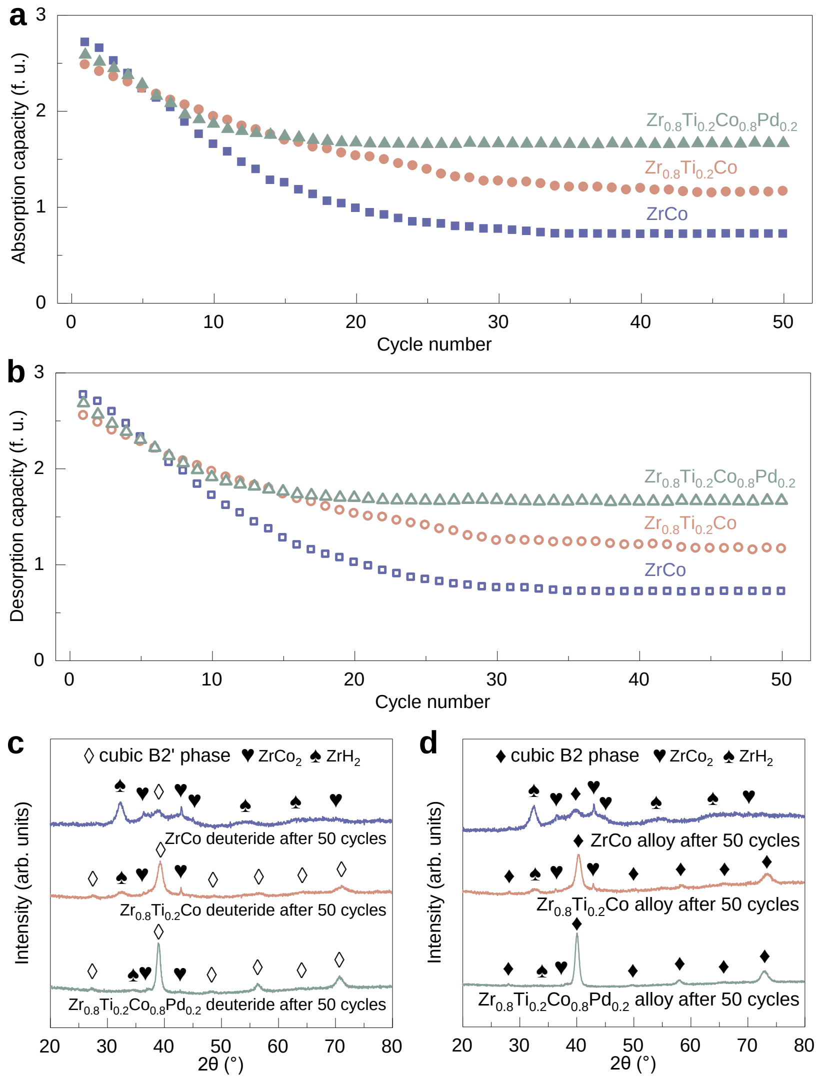

Supplementary Fig. 25 Cycling performance and characterization of ZrCo, $Zr_{0.8}Ti_{0.2}Co$ and $Zr_{0.8}Ti_{0.2}Co_{0.8}Pd_{0.2}$ alloys. Cyclic deuterium absorption **a** and desorption **b** capacities, and corresponding XRD patterns of deuterides **c** and alloys **d** at 50th cycle.

$Zr_{0.8}Ti_{0.2}Co_{0.8}Pd_{0.2}$ alloy exhibits the most stable cycling performance among ZrCo, $Zr_{0.8}Ti_{0.2}Co$ and $Zr_{0.8}Ti_{0.2}Co_{0.8}Pd_{0.2}$ alloys. Corresponding XRD patterns show that the variation of reaction pathway and the formation of disproportionation products lead to capacity attenuation.
